# Serum Sclerostin Is Associated with Peripheral and Central Systolic Blood Pressure in Pediatric Patients with Primary Hypertension

**DOI:** 10.3390/jcm10163574

**Published:** 2021-08-13

**Authors:** Piotr Skrzypczyk, Anna Ofiara, Michał Szyszka, Anna Stelmaszczyk-Emmel, Elżbieta Górska, Małgorzata Pańczyk-Tomaszewska

**Affiliations:** 1 Department of Pediatrics and Nephrology, Medical University of Warsaw, 02-091 Warsaw, Poland; aniaofi@gmail.com (A.O.); mpanczyk1@wum.edu.pl (M.P.-T.); 2 Department of Pediatrics and Nephrology, Doctoral School, Medical University of Warsaw, 02-091 Warsaw, Poland; michalszyszkaa@gmail.com; 3 Department of Laboratory Diagnostics and Clinical Immunology of Developmental Age, Medical University of Warsaw, 02-091 Warsaw, Poland; anna.stelmaszczyk-emmel@wum.edu.pl (A.S.-E.); elzbieta.gorska@uckwum.pl (E.G.)

**Keywords:** sclerostin, primary hypertension, children, adolescents, arterial damage, blood pressure

## Abstract

Recent studies showed the significance of the canonical Wnt/beta-catenin pathway and its inhibitor—sclerostin, in the formation of arterial damage, cardiovascular morbidity, and mortality. The study aimed to assess serum sclerostin concentration and its relationship with blood pressure, arterial damage, and calcium-phosphate metabolism in children and adolescents with primary hypertension (PH). Serum sclerostin concentration (pmol/L) was evaluated in 60 pediatric patients with PH and 20 healthy children. In the study group, we also assessed calcium-phosphate metabolism, office peripheral and central blood pressure, 24 h ambulatory blood pressure, and parameters of arterial damage. Serum sclerostin did not differ significantly between patients with PH and the control group (36.6 ± 10.6 vs. 41.0 ± 11.9 (pmol/L), *p* = 0.119). In the whole study group, sclerostin concentration correlated positively with height Z-score, phosphate, and alkaline phosphatase, and negatively with age, peripheral systolic and mean blood pressure, and central systolic and mean blood pressure. In multivariate analysis, systolic blood pressure (SBP) and height expressed as Z-scores were the significant determinants of serum sclerostin in the studied children: height Z-score (β = 0.224, (95%CI, 0.017–0.430)), SBP Z-score (β = −0.216, (95%CI, −0.417 to −0.016)). In conclusion, our results suggest a significant association between sclerostin and blood pressure in the pediatric population.

## 1. Introduction

It is estimated that arterial hypertension (AH) affects 3–5% of the pediatric population, and its frequency increases with the age of the children studied [1]. Today, in Western countries, with adverse lifestyle changes, the incidence of primary hypertension (PH) is increasing and is slowly becoming the dominant form of AH also in the developmental age [2]. PH is now believed to be not only cardiovascular disease but a multifaceted syndrome involving abnormal body fat distribution, overactive renin-angiotensin-aldosterone, sympathetic and immune systems, and premature aging (including early vascular aging) [3].

The importance of calcium-phosphate disorders, including vitamin D, in the development of AH and target-organ damage has become the subject of intensive research in recent years [4]. Although the recently published VITAL study (The Vitamin D and OmegA-3 Trial) did not demonstrate an impact of vitamin D supplementation on cardiovascular morbidity in adults [5], research is ongoing on the potential interplay between cardiovascular and bone health.

Sclerostin is a protein made up of 190 amino acids encoded by the *SOST* gene located on 17q12-21 chromosome, produced mainly by osteocytes, but also by chondrocytes, cementocytes, kidneys, liver, and vascular smooth muscle cells. Initially, it was thought that sclerostin belongs to the bone morphogenic protein (BMP) family [6]. However, it is now known that sclerostin, alongside the Dickkopf-1 protein (Dkk-1) is the most important inhibitor of the Wnt/beta-catenin pathway. Wnt ligands connect to the frizzled receptor and LRP5/6 (low-density lipoprotein receptor-related protein) coreceptor, stabilizing the beta-catenin structure, inducing its transfer to the nucleus, and affecting the transcription of genes encoding the proteins responsible for bone formation. Sclerostin combines with LRP5/6 coreceptors and inhibits the Wnt/beta-catenin pathway, which, in effect, reduces bone formation by inhibiting the formation of new osteoblasts and has a negative impact on the survival of already existing osteoblasts. Recent data indicate that sclerostin also stimulates osteoclast differentiation and increases bone resorption [7,8,9]. In 2019, the U.S. Food and Drug Administration (FDA) and European Medicines Agency (EMA) issued a positive opinion on the authorization of a monoclonal antibody against sclerostin (romosozumab) in postmenopausal women with osteoporosis and a high risk of fractures.

The Wnt/beta-catenin system might be the link between the bone and vascular system. Increased Wnt/beta-catenin activity was found in the experimental model of vascular calcification [10] and sclerostin expression was demonstrated in atherosclerotic plaques and calcifications within the human aorta [11]. The role of the Wnt/beta-catenin system and its endogenous inhibitors in circulatory system is unclear. Studies have shown both a positive [12,13] and negative [14,15] association, as well as no significant relationship [16,17] between sclerostin levels and arterial damage and cardiovascular risk in adults.

Considering the short duration of the disease and a small number of comorbidities, the population of children and adolescents with primary hypertension seems to be an ideal group to study the relationship between sclerostin, blood pressure, and subclinical arterial damage. Our study aims to assess serum sclerostin concentration in pediatric patients with PH and analyze its relationship with peripheral and central blood pressure, parameters of arterial damage, and parameters of calcium-phosphate metabolism.

## 2. Materials and Methods

### 2.1. Study Group

This single-center cross-sectional study included patients with PH treated in 2018–2020 in one tertiary center of pediatric nephrology. The criterion for inclusion in the study was confirmed AH following ESH recommendations [1]. In all patients referred to the center, we confirmed hypertension using ambulatory blood pressure monitoring. Only patients with elevated both office and 24 h ambulatory blood pressure were included in the final analysis. The exclusion criteria were: secondary forms of hypertension, the coexistence of known bone pathology, abnormalities in biochemical parameters of calcium-phosphate metabolism, diabetes mellitus, chronic inflammatory disease, or acute infectious disease. Based on available literature on sclerostin, assuming a delta of 4.5 (effect size: 0.50), a 0.05 alpha level, and 80% power, we estimated the sample size at 60 patients [13,14,16,18,19,20]; 20 age- and sex-matched healthy subjects were included in the control group. In the statistical analysis, the control group was smaller than the study group due to the comparable variance of key parameters in both groups.

Participants were included in the study consecutively from among the patients after considering inclusion and exclusion criteria to exclude selection bias. In the analyzed period (2018–2020), there were also 31 patients with white coat hypertension and seven patients with masked hypertension who were excluded from further analysis.

### 2.2. Ethical Issues

The authors obtained approval of the local Bioethical Committee before initiating the research (approval no. KB/58/2016, 15 March 2016). All procedures involving human participants were in accordance with the highest ethical standards of the institutional research committee and were performed according to the Declaration of Helsinki on the treatment of human subjects and its later amendments. Informed consent was obtained from all participants’ representatives and participants (≥16 years) before enrolling in the study.

### 2.3. Clinical Parameters

In all participants, the following clinical parameters were assessed: sex, age (years), body height (cm), body weight (kg), body mass index (kg/m^2^), antihypertensive medications taken, and duration of hypertension (months). Anthropometric parameters were compared with the standards for the local population and presented in the form of Z-score [21]. Overweight and obesity were defined according to WHO recommendations as a BMI Z-score above 1 and 2, respectively.

### 2.4. Serum Sclerostin

Serum sclerostin (ELISA enzyme-linked immunosorbent assay) was evaluated in all children studied using a kit from Biomedica Medizinprodukte GmbH, Vienna, Austria (BI-20492 Sclerostin ELISA). Blood samples were drawn on fasting from all the participants and thawed immediately after centrifugation at −84 °C.

### 2.5. Biochemical Parameters

All the participants had their calcium-phosphate metabolism evaluated on the basis of: serum concentrations of calcium (mg/dL), phosphate (mg/dL), 25-hydroxyvitamin D–25OHD (ng/mL), parathyroid hormone (pg/mL), and alkaline phosphatase activity (IU/L). In addition, the following biochemical parameters were evaluated: serum concentrations of creatinine (mg/dL), uric acid (mg/dL), total HDL- and LDL-cholesterol (mg/dL), and triglyceride (mg/dL). The estimated glomerular filtration rate (eGFR) was calculated in all patients (mL/min/1.73 m^2^) according to the revised 2009 Schwartz formula [22]. In line with local recommendations, 25OHD concentration was classified as severe deficiency (0–10 ng/mL), deficiency (>10–20 ng/mL), suboptimal (>20–30), optimal (>30–50 ng/mL), high (>50–100 ng/mL), and toxic (>100 ng/mL) levels [23]. Normal calcium (8.8–10.7 mg/dL), phosphate (2.8–5.6 mg/dL), parathyroid hormone (12–95 pg/mL), and alkaline phosphatase activity (45–515 IU/L) were derived from the manufacturer’s reference values.

### 2.6. Blood Pressure and Parameters of Arterial Damage

Each participant had their blood pressure measured three times, and the mean of the second and third measurements was taken for further analysis. Blood pressure measurements were performed oscillometrically (Welch Allyn Patient Monitor, Welch Allyn, Skaneateles Falls, NY, USA) according to ESH recommendations and were analyzed using pediatric normative values [24]. All the patients studied also had 24 h ambulatory blood pressure measurement performed (Oscar 2 Suntech, SunTech Medical Inc., Morrisville, NC, USA) according to the American Heart Association guidelines [25]. The device was programmed to perform measurement every 15 min between 7 a.m. and 10 p.m. and every 30 min between 10 p.m. and 7 a.m., giving 78 measurements per 24 h. We analyzed only reports with at least 50 readings per 24 h. The mean number of successful measures was 69.3 ± 4.5 readings (from 54 to 78 readings). Activity and resting periods were assessed according to the patient’s individual diaries. Systolic, diastolic, and mean pressures (SBP, DBP, MAP, respectively), blood pressure loads, and nighttime blood pressure dipping (DIP) were analyzed. DIP below 10% was considered as disturbed circadian rhythm [25]. For both office and ambulatory monitoring, the blood pressure cuff was chosen following ESH recommendations and the devices’ instructions. In all individuals, the middle upper-arm circumference was measured, and an appropriate cuff was selected from the cuffs available (Welch Allyn VSM: 20–26 cm, 25–34 cm, 32–43 cm, Oscar 2 Suntech: 17–25 cm, 23–33 cm, and 31–40 cm).

Arterial structure and function tests were performed using following methods: ultrasonographic examination of the common carotid artery (ALOKA Prosound Alpha 6, Hitachi Ltd., Tokyo, Japan)—common carotid artery intima media thickness (cIMT) (mm), Z-score [26], and common carotid artery local stiffness (E-tracking); applanation tonometry (Sphygmocor, ATCOR, Sydney, Australia)—central blood pressure, pulse wave analysis, and aortic (carotid-femoral) pulse wave velocity (aPWV) (m/s) Z-score [27]. The detailed description of the methods used was presented in the previous manuscripts of the research group [4,28]. In brief, arterial structure and function measurements were performed in a quiet room with ambient temperature (20 ± 5 °C) after 5 min of resting. Peripheral pressure waveforms were recorded from the radial artery at the right wrist (patient in sitting position, with back supported). Once 20 sequential waveforms had been acquired, the transfer function was used to generate the central aortic pressure waveform. aPWV was measured in a supine position and calculated as a difference in the carotid-to-femoral path length divided by the difference in R wave to the foot of the pressure wave taken from the superimposed ECG and pressure tracings. The distance was measured as the distance from the right carotid sampling site to the jugular notch, subtracted from the distance from the jugular notch to the right femoral sampling site in accordance with pediatric normative values by Reusz et al. [27]. Measurements of peripheral pressure waveform and aPWV were obtained three times in each subject, and the mean value of these measurements were taken for further analysis. cIMT was measured in a patient in a supine position using a manual method approximately 1 cm proximal to the carotid bulb on the distal carotid wall. Six determinations of cIMT—three on the left and three on the right side—were obtained and averaged. All arterial measurements were performed by a single experienced investigator (P.S.).

### 2.7. Statistical Analysis

The results were statistically analyzed using TIBCO Statistica 13.3 software (TIBCO Software Inc., Palo Alto, CA, USA). The numerical data obtained were presented as mean, standard deviation (SD), and interquartile range (IQR, Q1, Q3). The normality of variables was studied using the Shapiro–Wilk test. Normally distributed data were compared with Student *t*-test for independent groups and non-normally distributed data using the Mann–Whitney U test. The relationship between the two groups of variables was analyzed using Pearson correlation or Spearman rank correlation (depending on the distribution). Percentages in both groups were compared using the chi-square test and Fisher’s exact test. Multivariate analysis was performed using a general linear model. Parameters correlating with each other with r > 0.600 were excluded from the final model to avoid collinearity. The results of the multivariate analysis were presented with beta coefficients, confidence intervals (CI), and *p*-value. A *p*-value < 0.05 was considered statistically significant.

## 3. Results

### 3.1. Clinical, Laboratory Parameters, Blood Pressure, and Arterial Damage

Clinical and biochemical parameters in the PH and control groups are presented in Table 1, and data supporting the results can be found in Table S1. Patients with PH had significantly higher body weight, BMI, higher serum concentrations of uric acid and triglycerides, and lower concentration of HDL-cholesterol. In the PH group, 14 (23.3%) children were overweight, and 18 (30.0%) were obese. The duration of hypertension was 19.0 ± 25.4 (3–24) months. Twenty-five subjects were treated with antihypertensive medications and amlodipine was the most commonly used drug. The blood pressure and arterial parameters analysis revealed significantly higher office central and peripheral blood pressure and 24 h ambulatory blood pressure in the PH group (Table 2). Patients with PH were characterized by significantly higher cIMT, aPWV, larger diameter of common carotid artery, and slower time to maximal artery diameter.

### 3.2. Sclerostin and Parameters of Calcium-Phosphate Metabolism

**Figure 1:**
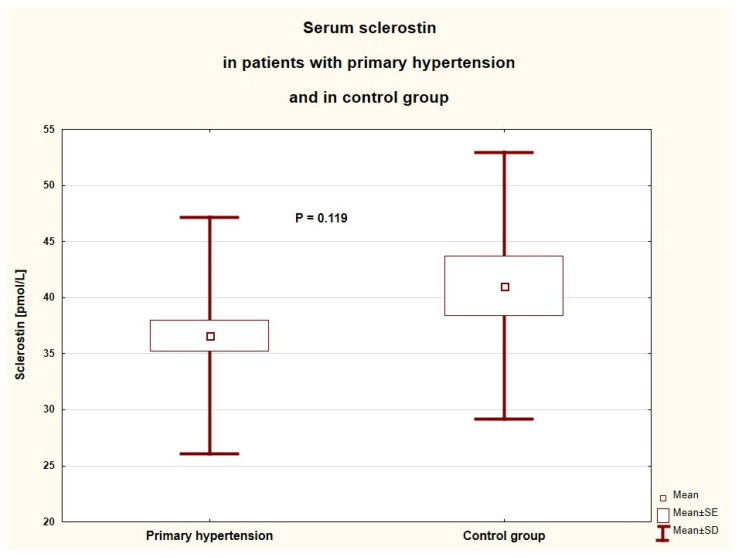
Serum sclerostin in children and adolescents with primary hypertension and in the control group (SE—standard error, SD—standard deviation).

The concentration of sclerostin and parameters of calcium-phosphate metabolism are shown in Table 3. The groups did not differ significantly in sclerostin concentration (Figure 1). All the patients studied had normal calcium, phosphate, parathyroid hormone, and alkaline phosphatase levels, but the serum calcium level was significantly higher in the PH group without any other differences between the groups. Severe deficiency and deficiency of vitamin D were found in more than half (56.3%) of the children studied. In children with PH, we showed no difference in serum sclerostin concentration between boys and girls (38.2 ± 10.0 vs. 34.1 ± 11.2 (pmol/L), *p* = 0.142) and between patients treated and untreated with antihypertensive agents (34.3 ± 10.6 vs. 38.3 ± 10.4 (pmol/L), *p* = 0.151).

### 3.3. Correlations of Sclerostin with Clinical and Biochemical Parameters, Blood Pressure, and Parameters of Arterial Damage

**Figure 2:**
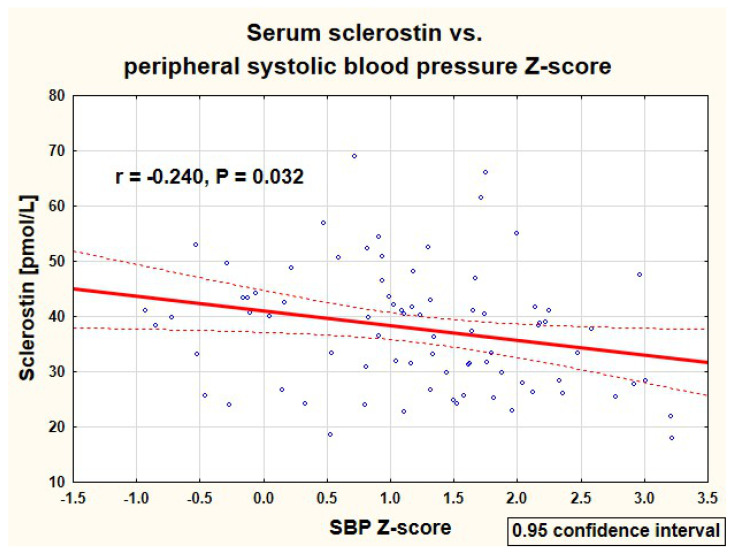
Correlation of serum sclerostin with peripheral systolic blood pressure Z-score in the studied children (SBP—peripheral systolic blood pressure).

**Figure 3:**
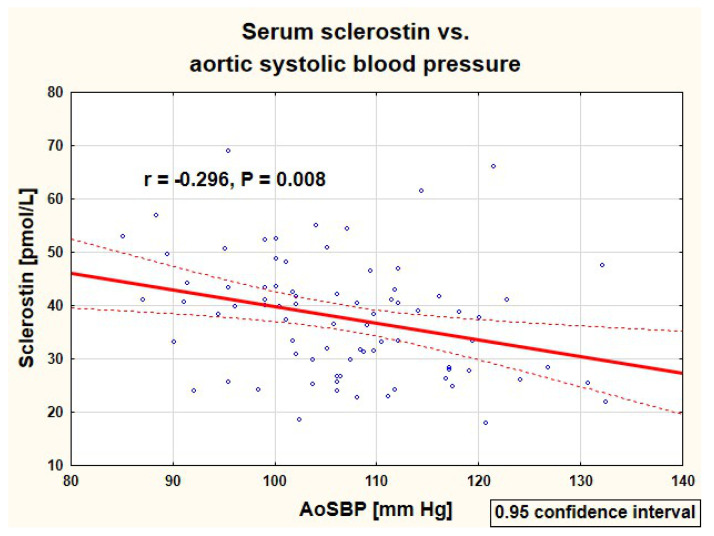
Correlation of serum sclerostin with central (aortic) systolic blood pressure in the studied children (AoSBP—aortic systolic blood pressure).

As there was no significant difference in serum sclerostin between PH and healthy children, both groups were analyzed together. Correlations of serum sclerostin concentration with analyzed clinical and biochemical parameters in 80 studied children are presented in Table 4. The serum sclerostin correlated positively with the height Z-score, serum phosphate, and alkaline phosphatase activity, and negatively with systolic and mean blood pressure. Negative correlations of serum sclerostin with peripheral and central systolic blood pressure are presented in Figure 2, Figure 3, respectively. There were no significant correlations of sclerostin and markers of arterial damage (aPWV (m/s): r = −0.065, *p* = 0.567, aPWV Z-score: r = −0.062, *p* = 0.584, AIx75HR (%): r = −0.053, *p* = 0.638, cIMT (mm): r = −0.084, *p* = 0.459, and cIMT Z-score: r = −0.092, *p* = 0.417).

The results of multivariate analysis (general linear model) are shown in Table 5. Systolic blood pressure and height expressed as Z-scores were the significant independent determinants of serum sclerostin in the studied children.

In a subanalysis of 35 untreated patients with primary hypertension, we found trends towards a negative correlation between serum sclerostin and peripheral systolic blood pressure (SBP) (mm Hg) (r = −0.323, *p* = 0.058), SBP Z-score (r = −0.299, *p* = 0.081), central systolic blood pressure (AoSBP) (mm Hg)) (r = −0.311, *p* = 0.069), central pulse pressure (AoPP (mm Hg)) (r = −0.292, *p* = 0.088), E-tracking pressure strain elasticity modulus (ET Ep) (r = −0.313, *p* = 0.067), and alkaline phosphatase (IU/L) (r = 318, *p* = 0.063).

## 4. Discussion

In our cross-sectional single-center study, we analyzed serum sclerostin in the group of pediatric patients with primary hypertension. Serum sclerostin did not differ significantly between hypertensive and normotensive children. Additionally, antihypertensive pharmacological treatment did not influence sclerostin concentration significantly. In the whole group of studied children, sclerostin correlated negatively with peripheral and central systolic and mean blood pressure; we also found significant positive association of sclerostin and calcium and phosphate metabolism parameters. In the multivariate analysis, systolic blood pressure and height, expressed as Z-scores, were the only significant determinants of serum sclerostin concentration. There was no significant correlation between parameters of arterial damage and serum sclerostin concentration.

Research studies from recent years suggested that Wnt/beta-catenin signaling system may be a crucial player in initiating and subsequently intensifying negative changes in the arteries. Its activation leads to the proliferation and apoptosis of vascular smooth muscle cells [29] and their transformation into osteoblast-like cells [30]. In addition, Wnt/beta-catenin pathway was shown to play a vital role in developing inflammation within the arterial wall by exacerbating the adhesion of monocytes to endothelium and promoting their passage through the vascular wall [31]. Therefore, it may be justified to hypothesize that inhibition of this signaling pathway by its inhibitors (sclerostin and DKK-1) in the arteries is a compensatory and protective mechanism [11,32].

Numerous studies in adults at high cardiovascular risk seem to support this hypothesis. Thambiah et al. demonstrated a negative relationship between DKK-1 and arterial stiffness (analyzed using the photoplethysmography method). However, the authors did not find such a correlation for sclerostin [16]. Gaudio et al. showed a negative correlation between cIMT and sclerostin concentrations in postmenopausal women with type 2 diabetes mellitus [14]. The same authors revealed a negative relationship between augmentation index and sclerostin concentration in 67 healthy adults [15]. However, one should not forget that some authors have shown a positive relationship between the concentration of circulating sclerostin and cIMT and arterial stiffness in adults [12,20,33]. The latter may indicate the complexity of the interaction between the Wnt/beta-catenin system and arterial wall.

In our cohort of pediatric patients with primary hypertension, we found no significant correlations between serum sclerostin and well-established indices of arterial wall damage (cIMT, aPWV, AIx75HR). Of note, to increase the precision of our analysis, we analyzed both aortic and local carotid damage. This negative finding can be partially explained by the relatively low severity of arterial lesions in children compared with adult patients. Large multicenter analyses revealed that subclinical arterial damage observed in children and adolescents are mainly adaptations of arterial wall to increased pressure (arteriosclerosis) and do not necessarily indicate the onset of atherosclerosis and calcification of the vascular wall [26,27,34]. On the other hand, in one of our previous studies, we have revealed a significant negative correlation between cIMT and serum fetuin level in pediatric PH patients [4]. Perhaps, other determinants of calcium-phosphate metabolism (e.g., calcification inhibitors), not Wnt/beta-catenin pathway, are involved in the earliest stages of hypertension-associated arterial damage.

Conversely, our results may indicate a protective effect of Wnt/beta-catenin inhibitors on the level of blood pressure in children. We showed numerous negative correlations between sclerostin and both central and peripheral blood pressure. We confirmed these univariate relationships in multivariate analysis. Additionally, a subanalysis of a small cohort of untreated PH subjects revealed a similar trend.

Of note, the relatively low duration of hypertension in our patients excluded advanced arterial lesions. In fact, notwithstanding significantly higher values of indexes of arterial stiffness (e.g., aPWV, AIx75HR, elastic modulus) in hypertensive than in normotensive patients, this difference might reflect the effect of higher blood pressure values. The absence of a significant relationship between serum sclerostin levels and indexes of arterial stiffness in the whole study population, as opposed to a significant negative correlation between sclerostin levels and systolic blood pressure Z-score at multivariate analysis, supports this interpretation. Thus, the results of this study should certainly be considered preliminary.

Contrary to our results, a positive correlation between serum sclerostin and systolic blood pressure (r = 0.262, *p* = 0.031) was demonstrated in kidney transplant adult patients [33]. In contrast, no significant association was seen in adults on hemodialysis [35] or with rheumatoid arthritis [18]. Whether sclerostin could be a good marker of cardiovascular morbidity and mortality remains an open question [36].

The protective role of sclerostin on circulatory system was suggested in a recent meta-analysis focused on cardiovascular events in patients treated with romosozumab. The findings of the meta-analysis indicate that the inhibition of sclerostin might elevate cardiovascular risk. Furthermore, the same author revealed that some variants of *SOST* gene were associated with significantly higher systolic blood pressure [37]. Interestingly, similarly to our results, systolic blood pressure, not diastolic blood pressure, seems to be associated with sclerostin action. Additionally, a pharmacovigilance analysis of the Food and Drug Administration Adverse Event Reporting System (FAERS) identified a potential signal for elevated risk of myocardial infarction, stroke, or cardiovascular death in patients treated with romosozumab [38]. Further research is needed to answer the nature of the interaction between the Wnt/beta-catenin system and the circulatory system. Nevertheless, these results warrant a rigorous evaluation of the cardiovascular safety of sclerostin inhibitors.

Some experimental and clinical data suggest the interaction of sclerostin and renin-angiotensin-system (RAS) in the formation of arterial damage. Sclerostin was found to inhibit angiotensin II-induced aortic aneurysm and atherosclerotic plaque formation in mice [39]. In addition, Mayer Jr et al. found a synergistic effect of sclerostin and angiotensin II receptor 1 polymorphism on arterial stiffening in the general adult population [40]. In our cohort, we have not analyzed concentrations of RAS components. Moreover, almost half of the subjects had already been treated with antihypertensive medications, which could have influenced possible RAS and sclerostin interaction.

In the analyzed group of patients, we did not find differences between sclerostin concentrations in boys and girls. Other authors found higher serum sclerostin in males in both children [19] and adults [6,16]. Higher concentrations of sclerostin in the male sex may be explained by higher bone mass. In the adult population, there was a positive relationship between sclerostin concentrations and age [6,16]. Increasing sclerostin levels with age may be due to an imbalance between bone formation and bone resorption processes [16]. Kirmani et al. analyzed in detail the relationship between sclerostin, age, bone age, densitometric parameters, and serological markers of bone turnover in children. The authors showed that sclerostin levels increase in boys up to 10 years of age, in girls up to 14 years of age, and then steadily decrease to start rising again around 20 years of age [19]. Since the vast majority of the children studied were in the second decade of life, our results (negative relationship between sclerostin and age) can be considered consistent with the results of Kirmani et al.

The same researchers found a positive relationship between sclerostin concentrations and markers of bone turnover: a marker of bone formation—N-terminal collagen propeptide type I (PINP) and a marker of bone resorption—C-terminal collagen telopeptide type I (CTX); however, there was no correlation between sclerostin and parathyroid and 25OHD levels [19]. Our group of children showed a positive relationship between height Z-score, serum phosphate, alkaline phosphatase activity and serum sclerostin concentration, which is most likely a reflection of larger bone mass and higher bone turnover in taller children.

Some limitations to our research need to be listed. This was a cross-sectional analysis; thus, final conclusions on the nature of the relationship between blood pressure and sclerostin cannot be drawn. Secondly, we did not analyze bone mass, which was found to have a substantial impact on serum sclerostin concentration. Our research did not examine the concentration of Dickkopf-1—another important inhibitor of Wnt/beta-catenin pathway. Additionally, despite the large study sample, analysis in subgroups (healthy children, treated/untreated patients) may have been subject to error due to small numbers. As many as 25 out of 60 analyzed hypertensive subjects had already been treated with antihypertensive medications, which could have influenced the final results. Antihypertensives might have masked some of the relationships, for example, between sclerostin and target-organ damage. Moreover, the short duration of arterial hypertension in our patients excluded advanced arterial lesions and masked a possible relationship with sclerostin observed in adult studies. Of note, despite many years of our clinical and research experience with the blood pressure monitors used, it should be emphasized that both these devices had been validated only in the adult population so far. The latter may have influenced the results, although it is essential that we tested both control and study groups with the same instruments using the same protocol.

## 5. Conclusions

Sclerostin, an endogenous inhibitor of Wnt/beta-catenin pathway, was found to be associated with cardiovascular damage in adults. In our research, serum sclerostin was not correlated to non-invasive indices of arterial damage but was inversely associated with systolic blood pressure. These results may suggest a significant association between sclerostin and blood pressure in the pediatric population and are consistent with studies on the cardiovascular safety of sclerostin inhibitors. There is a need for further studies at both experimental and clinical levels on the relationship between cardiovascular and Wnt/beta-catenin systems.

## Figures and Tables

**Table 1:** Clinical and biochemical data of the studied children.

Parameter	Children with Primary Hypertension	Healthy Children	*p*
Number of patients (n)	60	20	-
Age (years)	15.0 ± 2.9 (13.9–17.1)	14.4 ± 2.5 (12.6–16.8)	0.405
Gender (boys/girls)	37/23 (61.7%/38.3%)	9/11 (45%/55%)	0.205
Height (cm)	168.0 ± 16.2 (162–178)	164.9 ± 12.0 (158–174)	0.162
Height Z-score	0.50 ± 1.09 (−0.10–1.22)	0.54 ± 1.51 (−0.13–1.35)	0.689
Weight (kg)	71.4 ± 21.8 (60.5–82.7)	57.5 ± 15.4 (47.2–66.5)	0.010
Weight Z-score	1.20 ± 1.06 (0.39–1.95)	0.49 ± 1.14 (−0.27–1.53)	0.012
BMI (kg/m^2^)	24.7 ± 5.2 (20.92–28.36)	20.8 ± 3.8 (18.1–23.3)	0.003
BMI Z-score	1.15 ± 1.11 (0.39–2.13)	0.32 ± 0.91 (−0.49–1.13)	0.002
Antihypertensive medications (n)	25 (41.7%)	-	-
Calcium channel antagonists	19		
Angiotensin convertase inhibitors	8		
Beta-adrenolytics	3		
Diuretics	2		
Alfa-adrenolytics	2		
eGFR ac. to Schwartz (mL/min/1.73 m^2^)	101.9 ± 21.4 (88.8–113.1)	108.4 ± 20.8 (94.2–124.4)	0.192
Uric acid (mg/dL)	5.5 ± 1.4 (4.8–6.1)	4.5 ± 1.1 (3.8–5.5)	0.003
Total cholesterol (mg/dL)	159.9 ± 36.9 (136–171)	152.8 ± 29.5 (133–176)	0.509
HDL-cholesterol (mg/dL)	51.7 ± 14.4 (43–58)	57.7 ± 12.3 (49–64)	0.041
LDL-cholesterol (mg/dL)	88.6 ± 33.9 (66–98)	81.3 ± 25 (64–104)	0.505
Triglyceride (mg/dL)	98.4 ± 48.8 (63–121)	69.2 ± 26.3 (50–82)	0.010

BMI: body mass index; eGFR: estimated glomerular filtration rate; HDL: high-density lipoprotein; LDL: low-density lipoprotein.

**Table 2:** Blood pressure of the studied children.

Parameter	Children with Primary Hypertension	Healthy Children	*p*
Office oscillometric blood pressure			
SBP (mm Hg)	130.5 ± 11.6 (121–138)	119.0 ± 9.8 (109–126)	<0.001
SBP Z-score	1.43 ± 0.95 (0.93–2.12)	0.52 ± 0.79 (−0.17–1.13)	<0.001
DBP (mm Hg)	77.3 ± 10.8 (71–82)	67.7 ± 7.3 (65–74)	<0.001
DBP Z-score	1.65 ± 1.49 (0.88–2.28)	0.30 ± 0.91 (−0.14–1.06)	<0.001
Central blood pressure			
AoSBP (mm Hg)	109.6 ± 9.6 (102–117)	98.1 ± 8.1 (91–105)	<0.001
AoDBP (mm Hg)	79.2 ± 10.9 (72–84)	69.0 ± 7.2 (66–74)	<0.001
24 h ambulatory blood pressure			
SBP 24 h (mm Hg)	129.3 ± 9.6 (121–136)	114.8 ± 4.9 (112–117)	<0.001
DBP 24 h (mm Hg)	70.8 ± 7.7 (66–76)	64.4 ± 5.2 (62–68)	<0.001
MAP 24 h (mm Hg)	90.3 ± 7.7 (85–96)	81.1 ± 4.7 (78–85)	<0.001
SBPL 24 h (%)	44.0 ± 26.7 (27–63)	10.2 ± 6.8 (6–14)	<0.001
DBPL 24 h (%)	23.9 ± 21.0 (8–36)	7.3 ± 5.8 (2–11)	<0.001
SBP DIP (%)	10.3 ± 5.2 (8–13)	11.4 ± 3.4 (10–13)	0.383
DBP DIP (%)	15.3 ± 7.6 (11–20)	16.1 ± 3.7 (14–18)	0.642
Parameters of arterial damage			
cIMT (mm)	0.46 ± 0.07 (0.41–0.51)	0.41 ± 0.06 (0.31–0.42)	0.003
cIMT Z-score	1.47 ± 1.47 (0.45–2.41)	0.45 ± 1.08 (−0.29–0.68)	0.003
aPWV (m/s)	5.3 ± 1.0 (4.7–5.9)	4.6 ± 0.6 (4.2–5.0)	0.004
aPWV Z-score	−0.05 ± 1.17 (−0.95–0.71)	−0.88 ± 0.79 (−1.53–−0.33)	0.004
AIx75HR (%)	−2.1 ± 13.0 (−10.0–4.8)	−3.4 ± 13.0 (−9.2–3.3)	0.756
SEVR (%)	163.2 ± 41.2 (133–195)	155.6 ± 27.3 (137–169)	0.440
E-tracking beta	3.9 ± 3.3 (2.5–4.2)	3.7 ± 1.0 (3.1–4.5)	0.315
E-tracking Ep (kPa)	52.1 ± 42.3 (33–57)	44.9 ± 13.0 (34–55)	0.991
E-tracking AC (mm^2^/kPa)	1.3 ± 0.5 (0.9–1.6)	1.0 ± 0.2 (0.8–1.1)	0.016
E-tracking AIx (%)	−5.5 ± 17.2 (−11.9–−0.7)	−2.7 ± 6.1 (−4.5–0.4)	0.168
E-tracking PWVbeta (m/s)	4.1 ± 1.3 (3.4–4.4)	3.9 ± 0.6 (3.5–4.5)	0.934
E-tracking D_max (mm)	6.4 ± 0.7 (6.0–6.8)	5.7 ± 0.7 (5.1–6.3)	<0.001
E-tracking D_min (mm)	5.5 ± 0.7 (4.9–5.9)	4.9 ± 0.7 (4.3–5.4)	0.003
E-tracking DAT_max (ms)	128.5 ± 39.9 (105–140)	146.0 ± 41.8 (125–152)	0.026

SBP: systolic blood pressure; DBP: diastolic blood pressure; AoSBP: aortic (central) systolic blood pressure; AoDBP: aortic (central) diastolic blood pressure; SBPL: systolic blood pressure load; DBPL: diastolic blood pressure load; DIP: dipping; cIMT: common carotid artery intima-media thickness; aPWV: aortic pulse wave velocity; AIx75HR: augmentation index normalized to heart rate of 75 beats per minute; SEVR: subendocardial viability ratio; beta: stiffness index; Ep: pressure strain elasticity modulus; AC: arterial compliance; AIx: augmentation index; D_max: maximal diameter of the right common carotid artery; D_min: minimal diameter of the right common carotid artery; DAT_max: acceleration time to the right common carotid artery maximal diameter.

**Table 3:** Sclerostin and parameters of calcium-phosphate metabolism in studied children.

Parameter	Primary Hypertension	Healthy Children	*p*
Sclerostin (pmol/L)	36.6 ± 10.6 (27.3–42.7)	41.0 ± 11.9 (31.9–50.3)	0.119
Calcium (mg/dL)	10.0 ± 0.3 (9.8–10.2)	9.8 ± 0.3 (9.6–9.9)	0.005
Phosphate (mg/dL)	4.4 ± 0.7 (3.9–4.9)	4.4 ± 0.5 (4.0–4.7)	0.922
25OHD (ng/mL)	20.5 ± 7.8 (15.8–23.4)	20.3 ± 7.8 (15.8–22.3)	0.859
Severe vitamin D deficiency	3 (5.0%)	0 (0.0%)	0.580
Vitamin D deficiency	30 (50.0%)	12 (60.0%)	
Suboptimal vitamin D level	20 (33.3%)	7 (35.0%)	
Optimal vitamin D level	7 (11.7%)	1 (5.0%)	
Parathyroid hormone (pg/mL)	27.5 ± 12.7 (17–35)	22.9 ± 11.4 (13–31)	0.153
Alkaline phosphate (IU/L)	125.2 ± 67.8 (74–153)	154.3 ± 80.6 (91–213)	0.153

25OHD—25-hydroxyvitamin D.

**Table 4:** Significant correlations of sclerostin with clinical and biochemical parameters in the study group.

Analyzed Parameter	r	*p*
age (years)	−0.329	0.003
height Z-score	0.320	0.004
SBP (mm Hg)	−0.251	0.025
SBP Z-score	−0.240	0.032
MAP (mm Hg)	−0.226	0.044
AoSBP (mm Hg)	−0.296	0.008
AoMAP (mm Hg)	−0.253	0.024
phosphate (mg/dL)	0.323	0.003
alkaline phosphatase (IU/L)	0.462	<0.001

SBP: systolic blood pressure; MAP: mean arterial pressure; AoSBP: aortic (central) systolic blood pressure; AoMAP: aortic (central) mean blood pressure.

**Table 5:** Multivariate analysis of sclerostin determinants in the studied children.

Parameter	Beta	95% Confidence Interval	*p*
Height Z-score	0.224	(0.017–0.430)	0.034
SBP Z-score	−0.216	(−0.417–−0.016)	0.035
Phosphate (mg/dL)	0.219	(−0.002–0.441)	0.053
Age (years)	−0.170	(−0.396–0.055)	0.137

SBP: systolic blood pressure.

## Data Availability

Data used to support the findings of this study are included within the supplementary information files, Supplementary Table S1 (patient_data.xlsx).

## Supplementary Materials

The following are available online at https://www.mdpi.com/article/10.3390/jcm10163574/s1, Table S1: Patient data (patient_data.xlsx).
